# The implant treatment experience and knowledge scale: reliability and validity of a patient-reported outcome measure for dental implant therapy

**DOI:** 10.3389/froh.2026.1759348

**Published:** 2026-02-03

**Authors:** Engin Ozgur, Okan Cem Çırakoğlu, Emine Elif Alaaddinoglu, Nilgün Özlem Alptekin

**Affiliations:** 1 Department of Periodontology, Faculty of Dentistry, Baskent University, Ankara, Türkiye; 2 Department of Psychology, Faculty of Humanities, Cappadocia University, Nevsehir, Türkiye

**Keywords:** dental implants, patient reported outcome measures, patient satisfaction, quality of life, surveys and questionnaires, peri-implantitis, health knowledge, attitudes, practice

## Abstract

**Background:**

Despite increasing interest in patient-reported outcomes in implant dentistry, there is a lack of consensus on tool selection and outcome prioritization to assess patient-reported outcomes in implant dentistry. This study validates an instrument designed to support clinicians in evaluating patient experience and knowledge for personalized implant therapy.

**Methodology:**

Patients with dental implants that had been functioning for at least one year were included in this cross-sectional study. A five-point Likert questionnaire (Implant Treatment Experience and Knowledge Scale; ITEKS) was administered to measure patients’ perceived knowledge of peri-implant health/disease and their satisfaction with the treatment. The reliability of the questionnaire was analyzed using Cronbach's alpha. Exploratory factor analysis was used to examine the underlying structure of the questionnaire. Correlations between the OHIP-14, a widely used instrument for assessing oral health–related quality of life, and the administered scales were analyzed to examine convergent validity. The potential influence of patient-related factors (age, gender, education, source of information, and peri-implant diagnosis) was investigated.

**Results:**

A 28-item tool was used to assess dental implant treatment related awareness, satisfaction, post-treatment attitudes, and the etiology, treatment, and risk factors of peri-implantitis. The mean functional time of dental implants was 6.69 years. Psychometric properties were evaluated using exploratory factor analysis and Cronbach's alpha. Cronbach's alpha values for these tools were.864 and.779, respectively. Peri-implantitis was significantly associated with decreased patient satisfaction (*p* < .001). No statistically significant gender differences were observed for OHIP, Implant Treatment Experience Metric (ITEM), or Implant Patients Knowledge-Awareness Scale (IPKAS) scores (*p* > .05). Participants with advanced education exhibited significantly higher ITEM scores than those with basic education (*p* = .028). Furthermore, OHIP scores were significantly higher in the peri-implantitis group compared to both the peri-implant health and peri-implant mucositis groups (*p* < .001). Conversely, ITEM scores were significantly lower in the peri-implantitis group compared to the health and mucositis groups (*p* < .001).

**Conclusions:**

The instrument demonstrated methodological suitability for assessing patient-related outcomes in individuals undergoing implant treatment, as confirmed by reliability and validity tests. These results indicate that the ITEKS is a reliable and valid instrument for assessing patient experience in implant dentistry.

## 1 Introduction

Dental implants are a widely accepted treatment option for replacing missing teeth. They are highly effective in addressing tooth loss, with implant survival often serving as the primary measure of success (1). Studies with more than ten years of follow-up report implant survival rates of more than 95% (2–4). While these data provide a robust assessment of treatment outcomes, they do not capture patient-reported perspectives. Patient-reported outcome and experience measures (PROMs) complement traditional clinical endpoints by reflecting the patient's view of treatment benefits and satisfaction. Furthermore, as survival data require long-term follow-up, PROMs play an important role in assessing treatment outcomes in the shorter term and in strengthening both the internal validity (efficacy) and external validity (effectiveness) of dental implant studies (5).

The Oral Health Impact Profile (OHIP) is a widely validated instrument used to assess oral health–related quality of life across diverse patient populations, including dentate, partially edentulous, and implant-treated individuals (6–8). As a generic measure, the OHIP captures broad functional, psychological, and social impacts of oral conditions; however, it is not designed to assess treatment-specific experiences, expectations, or disease-related knowledge associated with dental implants and implant-supported restorations (6, 9). Accordingly, although the OHIP provides valuable information on general oral health impacts, it may lack sensitivity to implant-specific domains such as maintenance requirements, peri-implant disease awareness, and treatment-related satisfaction. In this context, the development of implant-focused patient-reported outcome measures can complement generic instruments by offering more precise insight into patient experience in implant dentistry (10–12). According to a systematic review by Boven et al., the instruments employed to measure treatment satisfaction varied widely, with few demonstrating reliability and validity (11). Another systematic review revealed that study designs and participant counts ranged from 18 to 300, with methods for assessing patient expectations differing significantly (13, 14). Accurately capturing the patient's perspective through PROMs necessitates measurement techniques with established methodological reliability and validity (15).

Dental implants and implant-supported restorations differ substantially from natural teeth and tooth-borne reconstructions; therefore, in addition to generic instruments, questionnaires with more precise and directly relevant statements are needed (8, 10, 16). However, the lack of consensus on tool selection and outcome prioritization challenges cross-study comparability (6, 12, 17, 18). A standardized, validated framework tailored to implant-specific outcomes (e.g., aesthetics, cost, maintenance, peri-implant diseases) could address this limitation (13, 19).

The purpose of this study is to evaluate the reliability and validity of the Implant Treatment Experience and Knowledge Scale (ITEKS), an instrument designed to assess patient satisfaction, attitudes, and knowledge regarding dental implants and peri-implant diseases in patients with functioning implants.

## 2 Methods

### 2.1 Study design

This cross-sectional study was conducted at Başkent University Faculty of Dentistry, Department of Periodontology, and was approved by the Ethics Committee for Non-Interventional Clinical Trials of Başkent University, Ankara, Turkey (D-KA19/31). The study adhered to the Declaration of Helsinki and followed the STROBE guidelines for cross-sectional studies (20).

### 2.2 Participant selection

Participants were recruited between January 2020 and November 2021. Adult patients who sought treatment at the Periodontology Clinic of Başkent University Faculty of Dentistry and had dental implants functioning for at least one year were included in the study. The inclusion criteria were as follows:

1. Age ≥18 years.
2. ASA status I or II.
3. Implants still in function.
4. Absence of repeated surgical interventions in the same area (e.g., soft tissue augmentation procedures, peri-implantitis treatment).

The exclusion criteria were as follows:

1. Metabolic bone diseases (e.g., osteoporosis, Paget's disease)
2. Current use of medications known to affect bone metabolism, such as high doses of antiresorptive agents (e.g., bisphosphonates, denosumab)
3. No response to phone calls or refusal to participate
4. Absence of any dental implants currently in function

### 2.3 Data collection

The university's database of implant patients, which was established in 2010, was used to evaluate the long-term effects of dental implant therapy. Patients were contacted sequentially from the earliest to the most recent records to maximize the observation period.

On the day of the examination, participants completed a questionnaire. To avoid bias, the questionnaire was completed before any clinical or radiographic examinations. In addition, demographic and clinical variables were collected, including age, gender, education level, presence of systemic diseases, smoking habit, and duration of implant function. A calibrated clinician (E.O.) performed the subsequent clinical and radiographic examinations. Intra-rater reliability was established, with all measurements exhibiting an Intraclass Correlation Coefficient (ICC >0.80). To investigate the relationship between peri-implant diagnoses and the instrument scores, key clinical parameters were recorded, including probing pocket depth (PD), clinical attachment loss (CAL), bleeding on probing (BOP), plaque index (PI), and keratinized mucosa width (KTW) (21, 22).

Participants were classified into three diagnostic categories: peri-implant health (PH), peri-implant mucositis (PM), and peri-implantitis (P-I) based on the clinical criteria established in the 2017 World Workshop on the Classification of Periodontal and Peri-Implant Diseases and Conditions (23). Moreover, to analyze the effect of age as a variable on the data, patients aged 44 and under were grouped as “young”, those between 45 and 64 as “middle age”, and those aged 65 and over as “advanced age” (24).

### 2.4 Questionnaire development process

#### 2.4.1 Item generation

The initial version of the ITEKS consisted of 31 statements covering dental implant knowledge, satisfaction, post-treatment attitudes, and peri-implantitis-related factors. To ensure a comprehensive assessment, an extensive review of the existing literature was conducted to identify and analyze relevant statements from various published instruments (10, 25). These items were carefully synthesized and refined to create a new, integrated instrument that addresses the most critical aspects of patient-reported outcomes. The goal was to establish a tool that covers all key domains, including aesthetics, maintenance, and risk factors, which generic tools often lack.

A 5-point Likert scale was used for participants to rate each statement, ranging from 1 = strongly disagree to 5 = strongly agree.

#### 2.4.2 Pilot testing

The preliminary questionnaire was piloted with the first 15 patients to ensure the clarity and comprehensibility of the items. During this phase, the research team observed the participants while they completed the tool, analyzing their reactions and time taken for each item. Following the completion of the questionnaire, semi-structured interviews were conducted with these patients to discuss their interpretation of each statement. The primary objective was to determine whether the participants’ understanding of the items matched the intended clinical and psychological constructs. In instances where a patient's interpretation deviated from the study's intention, minor wording adjustments and linguistic refinements were made to enhance clarity and reduce potential ambiguity.

#### 2.4.3 Item reduction

To ensure the internal reliability and structural integrity of the instrument, a systematic multi-step item reduction process was performed. Initially, items were screened based on their corrected item-total correlations. While a threshold of 0.30 was used as a statistical baseline for removal to ensure adequate homogeneity, the final decision to retain or exclude items was not based solely on mathematical indices. Clinical representativeness and content validity were also prioritized; items that demonstrated borderline statistical values but were deemed essential for capturing the full scope of patient experience and knowledge in implant dentistry were retained. This balanced approach ensured that the final version of the instrument maintained both high psychometric standards and practical clinical utility.

#### 2.4.4 Validation steps

Cronbach's alpha values were calculated to assess reliability. Two approaches were used to assess validity. Construct-related evidence was explored using exploratory multivariate techniques to examine the underlying structure of the item set. Given the early-stage development and clinically oriented aims of the instrument, Principal Component Analysis (PCA) was used as an exploratory approach to support item organization during initial instrument validation.

Prior to factor analysis, the reliability of the tool and the suitability of the data were assessed. An initial reliability analysis was performed on the 31-item ITEKS, resulting in the removal of three items with corrected item-total correlations below 0.30.

Convergent validity, the correlation of the tool with the widely accepted OHIP-14 defined by Slade et al. was examined (26, 27). The OHIP-14 uses 14 items to assess the impact of oral health on quality of life. It includes broad statements about previous negative experiences. While the OHIP-14 does not include implant-specific items, it provides a well-established generic framework for assessing oral health–related quality of life, allowing examination of convergent relationships between broad oral health impacts and implant-specific satisfaction and knowledge domains.OHIP-14 was analyzed using the total sum score, with higher scores indicating poorer oral health–related quality of life. Therefore, the possible relationship between biological complications, lower satisfaction, level of knowledge and OHIP-14 score was hypothesized. By combining with OHIP-14, participants responded to a total of 45 statements, providing a comprehensive assessment of their oral health-related quality of life.

### 2.5 Sample size calculation

A sample size calculation indicated that at least 192 patients (64 per diagnostic group) were required to detect moderate differences in mean numerical measures between groups with 90% power and a 5% error rate (effect size f = 0.26) (10). Moreover, Gómez-Polo et al. stated that they considered a ratio of 5–10 participants per item for tool validation analysis (15). With a sample size of 265 and a 28-item instrument, this yielded a ratio of approximately 9.46 participants per statement.

### 2.6 Statistical analysis

Data were analyzed using IBM SPSS Statistics software (version 26.0) and *p*-values < .05 were considered statistically significant. Demographic data and peri-implant diagnosis were presented with population size and frequency. Normally distributed data were expressed as mean ± standard deviation (SD), and One-way ANOVA was used to identify differences among subscales. Bonferroni *post-hoc* test was performed following ANOVA to determine which specific groups differed significantly from each other. Independent sample t-tests were used to analyze differences in OHIP scores based on gender and educational background.

Cronbach's alpha was used to assess the internal consistency of the tool. Exploratory factor analysis provided evidence regarding the structural organization of the questionnaire statements. Spearman correlation analysis was used to examine the relationship between the instruments and the OHIP-14.

Although individual Likert-scale items are ordinal in nature, composite scores derived from summated Likert items were treated as continuous variables for statistical analysis. This approach is widely accepted in psychometric and health research when scale scores comprise multiple items and demonstrate approximately normal distributions. Distributional assumptions were assessed using visual inspection of histograms and normality testing, which supported the use of parametric methods. In addition, the sample size was sufficiently large to justify the application of parametric tests under the central limit theorem (28).

## 3 Results

### 3.1 Descriptive statistics

A total of 903 patients were screened. 54 patients were excluded due to missing contact details, and 849 patients were called to invite them to participate in the study. Of these, 584 patients were excluded: 528 could not be contacted or declined the invitation, 23 had already lost their implants, 14 had undergone repeated surgical intervention in the same area, and 19 were excluded due to medication use, systemic disease, or other treatment-related causes. Ultimately, 265 participants (112 males, 153 females; mean age: 53.07 ± 12.21) with a total of 897 implants were included in this study to assess patient satisfaction, positive attitudes, and treatment knowledge. Tables 1, 2 show the demographics and peri-implant diagnosis of the participants.

**Table 1:** Demographic and peri-implant diagnosis data of the participants (*n* = 265).

Variables		Value (mean ± SD)	
Age		53.07 ± 12.21	
Function time of implants/years		6.69 ± 2.99	
Number of implants per patient		3.38 ± 2.51	
Characteristics	*n*	%	
Gender (*n* = 265)			
Female	153	57.7	
Male	112	42.3	
Educational Background (*n* = 265)			
Elementary	21	7.9	
High school	69	26.1	
College/University	175	66.0	
Smoking Habits (*n* = 265)			
Nonsmoker	145	54.7	
<10 a day	66	24.9	
>10 a day	54	20.4	
Peri-implant Diagnosis (Patient *n*:265)			
Peri-implant health	113	42.6	
Peri-implant mucositis	88	33.2	
Peri-implantitis	64	24.2	
Peri-implant Diagnosis (Implant *n*: 897)			
Peri-implant health	510	56.85	
Peri-implant mucositis	243	27.09	
Peri-implantitis	144	16.05	
Restorations (number of patients)	Implant survival	Previous implant lost	At risk of implant loss
Single-tooth implant restorations	98	1	2
Implant-supported bridges	108	4	28
Full-arch restorations	14	4	6

Implant survival: patients with no history of implant loss; Previous implant loss: patients who had experienced implant loss; At risk of implant loss: patients with implants diagnosed with a poor or hopeless prognosis.

**Table 2:** Comparison of demographic data between peri-implant conditions.

Variables	PH (*n* = 113)		PM (*n* = 88)		P-I (*n* = 64)		*p*
Age (mean ± SD)	49.16 ± 12.49		54.80 ± 11.16a		57.59 ± 11.10a		**<0.001^a^**
Function time of implant in years (mean ± SD)	6.37 ± 2.67a		6.19 ± 2.99a		7.93 ± 3.22		**<0.001^a^**
Number of Teeth (mean ± SD)	24.82 ± 4.32		20.11 ± 5.74		13.91 ± 8.46		**<0.001^a^**
	*n*	%	*n*	%	*n*	%	*p*
Gender							*p* = 0.474^b^
Male	47	17.7	34	12.8	31	11.7	
Female	66	24.9	54	20.4	33	12.5	
Information source							*p* = 0.859^b^
Dentist	82	30.9	61	23.0	46	17.4	
TV/internet	9	3.5	4	1.6	6	2.3	
Family/Friends	16	39.0	17	6.4	8	3.0	
Smoking							*p* = 0.101^b^
No smoking	66	24.9	46	17.4	33	12.5	
Less than 10	32	12.1	22	8.3	12	4.5	
More than 10	15	5.7	20	7.5	19	7.2	

Mean ± SD, mean ± standard deviation; PS, peri-implant health; PM, peri-implant mucositis; P-I, peri-implantitis, *p < 0.05, ^a^One way ANOVA ^b^Chi-square analysis.

Values in bold indicate statistical significance (*p* < 0.05).

### 3.2 Exploratory factor analysis and reliability analysis

After extraction, three items were removed, and the remaining 28 items were subjected to further analysis. The removed items included statements regarding the cost of treatment (“Dental implants are an expensive treatment option”), biological risks (“A dental implant can be rejected by the bone”), and perceived patient knowledge (“I have sufficient knowledge regarding potential problems that may develop around the implant”). The suitability of the data for factor analysis was evaluated using the Kaiser-Meyer-Olkin (KMO) measure and Bartlett's Test of Sphericity. The KMO value was.867, and Bartlett's test was significant [*χ*^2^ (378) = 3,448.153, *p* < .001], indicating that the data were appropriate for factor analysis. Additionally, the sampling adequacy of individual items was assessed using an anti-image correlation matrix.

Principal Component Analysis (PCA) was applied to the 28 remaining items. An oblique rotation (oblimin) was used because the underlying constructs measured by the questionnaire were theoretically expected to be correlated, given the conceptual overlap between patient knowledge, awareness, and satisfaction domains. Allowing correlations between factors was therefore considered more appropriate than assuming orthogonality. The rotation converged after 3 iterations, indicating a stable factor solution. The number of factors was determined based on eigenvalues greater than 1.00 and examination of factor loadings. The communalities (h^2^) after extraction ranged from 0.15 to 0.75, with the majority of items showing acceptable values (>0.30), suggesting that the extracted factors explained a substantial proportion of item variance.

Exploratory analysis of the item set revealed a multidimensional empirical structure, with items clustering into two interpretable components reflecting treatment experience and knowledge–awareness domains. The final PCA with the 28 items yielded a two-factor solution. Factor loadings, eigenvalues, percentages of variance explained, and internal consistency coefficients were presented in Table 3.

**Table 3:** Exploratory factor analysis of “ITEKS””.

Statements	Mean ± SD	Factor 1 (ITEM)	Factor 2 (IPKAS)
S-1 I am satisfied with my dental implants	3.97 ± 0.96	0.865	
S-2 I recommend implant treatment to people who have lost their teeth	3.99 ± 0.86	0.853	
S-3 I think it's worth the money I paid.	3.94 ± 0.98	0.845	
S-4 The implant treatment met my expectations.	4.02 ± 1.05	0.844	
S-5 If I had to, I would have implants again.	3.87 ± 1.02	0.784	
S-6 Dental implant is the best treatment option for missing teeth.	3.97 ± 0.94	0.757	
S-7 My doctor informed me extensively about implant treatment.	4.04 ± 0.92	0.716	
S-8 Implants are as aesthetic as natural teeth.	3.85 ± 0.85	0.664	
S-9 I can use my dental implants for lifetime	3.32 ± 1.01	0.542	
S-10 The disease that may develop around the implant is easy to treat	2.84 ± 0.95	0.537	
S-11 Implants do not cause any side effects or diseases.	2.78 ± 1.04	0.512	
S-12 My doctor did not inform me enough about the diseases that may develop around the implant.	3.63 ± 1.12	0.486	
S-13 Implants last longer than natural teeth.	3.16 ± 1.04	0.462	
S-14 The problem that develops around the implant is the fault of the dentist.	3.51 ± 0.98	0.458	
S-15 Implants can be applied to all patients for the treatment of tooth deficiencies.	2.95 ± 1.01	0.430	
S-16 Dental caries is not a threat to implants.	3.24 ± 1.06	0.430	
K-1 Gum disease is a serious risk for implant health.	3.62 ± 0.99		0.804
K-2 Individuals who have had gum disease before have a higher risk of developing problems around the implant.	3.23 ± 0.92		0.682
K-3 Bacteria in the mouth compromise the health of the tissues around the implant.	3.55 ± 0.90		0.673
K-4 Implant health deteriorates in individuals with poor oral care.	4.05 ± 0.69		0.635
K-5 Smoking is an important cause of deterioration of implant health.	3.52 ± 0.87		0.588
K-6 Diabetics have a higher risk of developing problems around the implant.	3.37 ± 0.75		0.549
K-7 Individuals with thin bones develop more problems around the implant.	3.49 ± 0.89		0.465
K-8 The implant does not need to be cleaned as much as natural teeth.	4.09 ± 0.86		0.455
K-9 If the disease develops around the implant, the implant may be lost.	3.73 ± 0.87		0.448
K-10 Disease can develop around the implant.	3.73 ± 0.76		0.429
K-11 Regular control is important for implant health.	4.23 ± 0.68		0.419
K-12 I've heard/know about peri-implantitis.	1,96 ± 1.21		0.333
Items Excluded from the Final Instrument			
Dental implants are an expensive treatment option.			
A dental implant can be rejected by the bone.			
I have sufficient knowledge regarding potential problems that may develop around the implant.			
Eigenvalue		7.456	3.873
% of variance		26.628	13.833
Cronbach-α		.864	.779

Mean ± SD, mean ± standard deviation; Cronbach-α value of ITEKS:.855, % of variance: 40.461.

Factor 1 (Implant Treatment Experience Metric—ITEM): This factor comprised 16 items related to patient satisfaction and positive attitudes. It accounted for 26.63% of the total variance, with a Cronbach's alpha of.864.

Factor 2 (Implant Patients Knowledge-Awareness Scale—IPKAS): This factor included 12 items concerning risk factors, etiology, prevention, and peri-implant diseases. It explained 13.83% of the total variance, with a Cronbach's alpha of.779.

Although item K-12 (“I've heard/know about peri-implantitis”) exhibited a factor loading below 0.40, it was retained based on content-validity considerations. This item captures a clinically essential indicator of baseline awareness and enhances the instrument's sensitivity across varying levels of patient health literacy. Its retention was therefore guided by conceptual relevance rather than statistical optimization alone.

Together, the two factors accounted for 40.46% of the total variance. Overall Cronbach's alpha for the ITEKS was.855, indicating high internal consistency. The frequency distributions of the 5-point Likert scale responses for each item of the ITEKS are presented in Table 4.

**Table 4:** Frequency distribution of patient responses for each item of the ITEKS (*N* = 265).

Item number	Strongly disagree *n* (%)	Disagree *n* (%)	Neutral *n* (%)	Agree *n* (%)	Strongly agree *n* (%)
S1	8 (3.0%)	21 (7.9%)	16 (6.0%)	147 (55.5%)	73 (27.5%)
S2	7 (2.6%)	10 (3.8%)	28 (10.6%)	153 (57.7%)	67 (25.3%)
S3	10 (3.8%)	17 (6.4%)	26 (9.8%)	137 (51.7%)	75 (28.3%)
S4	13 (4.9%)	17 (6.4%)	14 (5.3%)	128 (48.3%)	93 (35.1%)
S5	14 (5.3%)	16 (6.0%)	27 (10.2%)	141 (53.2%)	67 (25.3%)
S6	4 (1.5%)	18 (6.8%)	44 (16.6%)	116 (43.8%)	83 (31.3%)
S7	7 (2.6%)	18 (6.8%)	13 (4.9%)	147 (55.5%)	80 (30.2%)
S8	6 (2.3%)	18 (6.8%)	30 (11.3%)	168 (63.4%)	43 (16.2%)
S9	15 (5.7%)	37 (14.0%)	85 (32.1%)	104 (39.2%)	24 (9.1%)
S10	24 (9.1%)	67 (25.3%)	109 (41.1%)	58 (21.9%)	7 (2.6%)
S11	24 (9.1%)	94 (35.5%)	79 (29.8%)	53 (20.0%)	15 (5.7%)
S12	12 (4.5%)	45 (17.0%)	27 (10.2%)	126 (47.5%)	55 (20.8%)
S13	23 (8.7%)	42 (15.8%)	85 (32.1%)	99 (37.4%)	16 (6.0%)
S14	5 (1.9%)	43 (16.2%)	66 (24.9%)	115 (43.4%)	36 (13.6%)
S15	11 (4.2%)	63 (23.8%)	71 (26.8%)	91 (34.3%)	29 (10.9%)
S16	18 (6.8%)	73 (27.5%)	96 (36.2%)	61 (23.0%)	17 (6.4%)
K1	4 (1.5%)	40 (15.1%)	57 (21.5%)	117 (44.2%)	47 (17.7%)
K2	5 (1.9%)	47 (17.7%)	124 (46.8%)	61 (23.0%)	28 (10.6%)
K3	1 (0.4%)	37 (14.0%)	79 (29.8%)	112 (42.3%)	36 (13.6%)
K4	3 (1.1%)	6 (2.3%)	21 (7.9%)	179 (67.5%)	56 (21.1%)
K5	3 (1.1%)	32 (12.1%)	82 (30.9%)	121 (45.7%)	27 (10.2%)
K6	2 (0.8%)	20 (7.5%)	138 (52.1%)	87 (32.8%)	18 (6.8%)
K7	4 (1.5%)	30 (11.3%)	93 (35.1%)	108 (40.8%)	30 (11.3%)
K8	1 (0.4%)	18 (6.8%)	28 (10.6%)	127 (47.9%)	91 (34.3%)
K9	5 (1.9%)	18 (6.8%)	62 (23.4%)	139 (52.5%)	41 (15.5%)
K10	2 (0.8%)	22 (8.3%)	45 (17.0%)	173 (65.3%)	23 (8.7%)
K11	0 (0.0%)	8 (3.0%)	14 (5.3%)	153 (57.7%)	90 (34.0%)
K12	137 (51.7%)	53 (20.0%)	38 (14.3%)	23 (8.7%)	14 (5.3%)

### 3.3 Convergent validity

To assess the convergent validity of the ITEKS, Spearman's rank-order correlations were calculated between its subscales (ITEM and IPKAS) and the Oral Health Impact Profile (OHIP-14), a well-established measure of patient-reported outcome measures (PROMs) in dentistry. It was hypothesized that ITEKS subscales would show significant correlations with OHIP-14, reflecting shared constructs related to implant treatment outcomes.

The correlation analyses of clinical parameters and tools were presented in Tables 5, 6. As expected, both ITEKS subscales were significantly correlated with OHIP-14. Specifically, ITEM showed a negative correlation with OHIP-14 (*p* < .01), indicating that higher satisfaction with implant treatment was associated with lower oral health-related impairment. Conversely, IPKAS exhibited a positive correlation with OHIP-14 (*p* < .01), suggesting that greater knowledge and awareness of implant-related factors were associated with increased scores of oral health impacts.

**Table 5:** Correlation analysis of clinical parameters and instruments.

Clinical parameters	CAL	BOP	PI	KTW	REC	OHIP-14	ITEM	IPKAS
PD	0.92**	0.61**	0.48**	-0.32**	0.38**	0.23**	-0.13**	0.01
CAL		0.61**	0.50**	−0.33**	0.66**	0.17**	−0.08	0.01
BOP			0.81**	−0.26**	0.25**	0.28**	−0.07	0.02
PI				−0.30**	0.26**	0.27**	−0.05	0.02
KTW					−0.19**	−0.22**	0.06	−0.16**
REC						0.01	0.06	0.01

Correlations are based on Spearman's rank-order method. **p < .01, *p < .05 CAL, clinical attachment loss; BOP, bleeding on probing; PI, plaque index; KTW, keratinized tissue width; REC, recession.

**Table 6:** Correlation analysis of the instruments and number of implants.

Variables	Number of Implant	OHIP-14	ITEM	IPKAS
Number of Implant		0.27**	−0.02	0.03
OHIP-14			−0.20**	0.23**
ITEM				0.07
IPKAS				

Correlations are based on Spearman's rank-order method. **p < .01, *p < .05.

Regarding the extent of therapy, the mean number of implants per patient was 3.38 ± 2.51. Analysis of the relationship between the number of implants and instrument scores (Table 6) revealed a significant correlation with OHIP-14 scores (*p* < .01). However, no statistically significant correlations were observed between the number of implants and the ITEM or IPKAS scores (*p* > .05), suggesting that these specific dimensions of patient knowledge and satisfaction may be independent of the quantity of implants received.

### 3.4 Comparison of groups

The distribution of instrument scores across the peri-implant diagnostic categories and age groups is presented in Tables 7, 8, respectively. Peri-implantitis was found to be significantly associated with decreased patient satisfaction (*p* < .001).

**Table 7:** Comparison of the instruments scores between peri-implant conditions (*n* = 265).

Instruments	PH (*n* = 113)	PM (*n* = 88)	P-I (*n* = 64)	*p*
OHIP-14	20.23 ± 6.09a	22.82 ± 9.51a	27.85 ± 9.59	**<0.001**
Implant treatment experience metric	3.73 ± 0.41a	3.57 ± 0.56a	3.25 ± 0.87	**<0.001**
Implant patients knowledge-awareness	3.51 ± 0.43	3.50 ± 0.44	3.66 ± 0.57	0.067

All data presented with mean ± standard deviation, One-way ANOVA test was used to analyze parametric variables.

Values in bold indicate statistical significance (*p* < 0.05).

**Table 8:** Distribution of the instrument scores by age groups.

Variables	Young individuals (*n* = 65)	Middle age (*n* = 151)	Advanced age (*n* = 49)	*p*
Peri-implant Health	39 (% 60)	62 (% 41.1)	12 (% 24.5)	**<0.001**
Peri-implant Mucositis	17 (% 26.2)	52 (% 34.4)	19 (% 38.8)	0.231
Peri-implantitis	9 (% 13.8)^a^	37 (% 24.5)^a,b^	18 (% 36.7)^b^	**0.018**
Number of implant	2.27 ± 1.57	3.69 ± 2.62	3.89 ± 2.77	**<0.001**
Function time	5.39 ± 2.39	6.88 ± 3.05	7.79 ± 2.99	**<0.001**
OHIP-14	22.09 ± 9.90	23.44 ± 8.73	22.49 ± 7.05	0.540
Implant treatment experience metric	3.58 ± 0.55	3.52 ± 0.67	3.67 ± 0.58	0.365
Implant patients knowledge-awareness	3.59 ± 0.51	3.52 ± 0.47	3.54 ± 0.44	0.633

All data presented with mean ± standard deviation, ANOVA was utilized for multiple comparisons, followed by the Bonferroni *post-hoc* test for pairwise comparisons.

Values in bold indicate statistical significance (*p* < 0.05).

To examine potential gender-based variations in the measured outcomes, independent samples t-tests were conducted. Independent sample t-tests revealed no statistically significant differences between genders for OHIP, ITEM, and IPKAS scores (*p* > .05).

When examining the relationship between educational background and tool scores, independent sample t-tests showed that participants with advanced education exhibited significantly higher ITEM scores compared to those with basic education (*p* = .028).

One-way ANOVA analysis examining the relationship between peri-implant diagnosis and tool scores demonstrated that OHIP scores were significantly higher in the peri-implantitis group compared to both peri-implant health and peri-implant mucositis groups (*p* < .001). Although the peri-implantitis group exhibited numerically higher IPKAS scores, no significant differences were observed among diagnostic groups (*p* > .05). However, ITEM scores were significantly lower in the peri-implantitis group compared to peri-implant health and peri-implant mucositis groups (*p* < .001).

## 4 Discussion

The purpose of this study was to evaluate the suitability of the ITEKS for assessing PROMs in implant dentistry. Currently used instruments such as OHIP-14, OIDP, GOHAI, and OHRQoL may contain confusing statements and are inadequate for specifically assessing PROMS in dental implant patients (8, 10, 16). Answering generic questions can be challenging, as patients may struggle to relate them directly to their treatment (16). Their statements evaluate general changes in well-being and generic instruments such as the OHIP capture broad changes in oral health–related quality of life but may lack sensitivity to treatment-specific experiences and knowledge domains relevant to implant therapy (6). Therefore, these instruments may lack the sensitivity necessary to accurately evaluate outcomes related to the location of the dental implant. Additionally, responses to broad questions can be influenced by many external factors, deviating from the primary issue and failing to provide meaningful results (7). This can make it difficult for researchers to interpret the data and identify relationships or underlying causes.

Cronbach's alpha, a widely used technique for assessing internal consistency, was calculated (5, 6, 10, 14). The Cronbach's alpha coefficient for the ITEKS questionnaire, which included 28 statements, was.855. According to the literature, a Cronbach's alpha value greater than.70 is considered to indicate good reliability (15). The ITEM and the IPKAS instrument demonstrated reliability with Cronbach's alpha values of.864 and.779, respectively. These results demonstrated adequate internal consistency and reliability.

The exploratory psychometric findings indicate that the ITEKS exhibits a coherent multidimensional empirical structure, with two components reflecting implant treatment experience and knowledge–awareness domains. The two-component solution accounted for 40.46% of the total variance, a proportion comparable to that reported for multidimensional patient-reported outcome measures addressing complex experiential and cognitive constructs in oral health research. Given the multifaceted nature of patient satisfaction, attitudes, and disease-related knowledge, a moderate level of explained variance is expected and does not detract from the instrument's clinical relevance. As the primary aim of this analysis was exploratory, these findings provide preliminary construct-related evidence, and further validation using confirmatory factor analysis in independent samples is warranted.

The OHIP-14 is recognized as a reliable and valid tool for assessing the impact of oral health on quality of life (6, 26). In addition to factor analysis, the OHIP-14 was employed to evaluate the convergent validity of the instruments through correlation analyses with patient-reported quality of life (29). A negative correlation was found between OHIP-14 scores and scores on the ITEM (*p* < .01). This suggests that individuals with higher OHIP-14 scores, indicating more negative oral health experiences, reported lower levels of satisfaction. Similarly, a statistically significant correlation was observed between the keratinized mucosa width and both the OHIP-14 and the IPKAS (22). Insufficient keratinized tissue around dental implants is associated with increased plaque accumulation, inflammation, and brushing discomfort, which may explain these findings (30, 31).

The OHIP-14 is a generic oral health–related quality of life instrument with extensive validation across heterogeneous patient populations. Its strength lies in capturing broad functional and psychosocial impacts of oral conditions; however, it is not designed to assess treatment-specific experiences, expectations, or disease-related knowledge. In this context, the ITEKS does not replace generic PROMs such as the OHIP-14 but complements them by providing implant-specific insight, thereby enhancing the precision and clinical interpretability of patient-reported outcomes in implant dentistry.

However, patients demonstrated a positive correlation between OHIP-14 and the prevalence of peri-implantitis diagnosis. Participants in the peri-implant disease groups had significantly higher OHIP-14 scores, reflecting more negative experiences, which corresponded to lower satisfaction scores. While knowledge scores did not differ significantly between groups, participants in the peri-implantitis group tended to have higher knowledge scores, which correlated positively with their OHIP-14 scores and peri-implant diagnosis. This finding suggests that adverse experiences with peri-implant disease may lead to higher levels of awareness and knowledge (7, 32, 33).

Clinicians typically focus on the surgical and prosthodontic aspects of treatment, which are related to various technical parameters (1–3). To achieve the next level of success, clinicians may be overlooking the importance of evaluating PROMS sufficiently (12). This oversight can lead to unrealistic patient expectations, such as regarding aesthetics, treatment longevity, or responsibilities related to the treatment (13). Additionally, a lack of awareness about potential health issues may threaten the success of implant therapy (34).

Evaluating PROMS allows clinicians to better understand a patient's mindset and identify any misunderstandings or gaps in their understanding of the treatment (5, 14). This enables clinicians to tailor treatment plans accordingly, and in some cases, even adjust the treatment protocol. It also helps clinicians allocate more time to address misunderstandings and manage unrealistic expectations (17).

Several limitations should be acknowledged. First, the cross-sectional design precludes causal inferences and limits the ability to assess changes in patient-reported outcomes over time. Longitudinal validation is therefore necessary to evaluate the sensitivity and long-term responsiveness of the instrument, particularly as biological complications emerging over time may impact patient satisfaction and knowledge (35). Second, the study was conducted in a single university-based setting where patients receive intensive periodontal care and education (10). This environment may result in higher patient awareness than routine private practice, potentially limiting the generalizability of the findings. Third, while the sample size was adequate for psychometric analyses, cultural and healthcare-system–specific factors may influence patient perceptions, necessitating external validation in more diverse populations. Additionally, self-reported measures are inherently subject to response and recall bias. Finally, although the ITEKS itself comprises 28 items and demonstrated good reliability and validity, the combined administration of the ITEKS and the OHIP-14 (totaling 45 items) may have increased respondent burden and could limit feasibility in busy clinical settings (5).

Future research should prioritize longitudinal studies with larger, demographic-specific cohorts to address current limitations and provide more robust findings regarding the instrument's external validity. There is a clear opportunity to explore the development of shorter versions of the questionnaire to enhance its utility in high-volume clinical environments. Moreover, the integration of these instruments into the standard clinical workflow will be valuable for personalized care. From this perspective, clinicians can utilize the ITEKS to assess patients’ awareness and knowledge levels, determining whether additional information regarding potential risks is required or if specific interventions are needed to motivate patients toward preventing future biological complications (36).

## 5 Conclusion

Reliability analyses and exploratory validity evidence support the methodological suitability of the ITEKS as an initial, implant-specific instrument for assessing patient-reported outcomes in implant dentistry.

## Data Availability

The raw data supporting the conclusions of this article will be made available by the authors, without undue reservation.
